# Phase III study of HR-positive/HER2-negative/lymph node-positive breast cancer non-responsive to primary chemotherapy: a randomized trial

**DOI:** 10.1038/s41523-023-00553-y

**Published:** 2023-06-21

**Authors:** Yang Yang, Yingjian He, Zhaoqing Fan, Xue Chen, Yiqiang Liu, Chao Zhang, Hongchuan Jiang, Xin Wang, Xiang Wang, Fei Xie, Shu Wang, Bin Luo, Hua Kang, Tao Wang, Zefei Jiang, Peng Yuan, Binhe Xu, Ling Xu, Yinhua Liu, Jinfeng Li, Yuntao Xie, Tianfeng Wang, Tao Ouyang

**Affiliations:** 1 Key Laboratory of Carcinogenesis and Translational Research (Ministry of Education), Breast Center, Peking University Cancer Hospital & Institute, Beijing, China; 2 Beijing Chao Yang Hospital, Breast Disease Department, Beijing, China; 3 Cancer Hospital, Chinese Academy of Medical Sciences, Breast Cancer Department, Beijing, China; 4 Peking University People’s Hospital, Breast Cancer Center, Beijing, China; 5 Beijing Tsinghua Changgeng Hospital, Breast Disease Department, Beijing, China; 6 Xuan Wu Hospital Capital Medical University, Breast Disease Department, Beijing, China; 7 307 Hospital of PLA, Breast Cancer Department, Beijing, China; 8 Peking University First Hospital, Breast Cancer Center, Beijing, China

**Keywords:** Randomized controlled trials, Cancer therapy

## Abstract

There are few studies focus on post-neoadjuvant treatment in hormone receptor-positive (HR+)/human epidermal growth factor receptor 2-negative (HER2-)/lymph node-positive (LN+) breast cancer, a multi-center, open-label, randomized, controlled phase III trial was conducted to evaluate pathological response-guided non-cross-resistant adjuvant chemotherapy in patients with HR+/HER2-/LN+ breast cancer who were non-responsive to primary chemotherapy. Patients received four cycles of non-cross-resistant adjuvant chemotherapy plus endocrine therapy (ET), or ET alone. Forty patients responsive to neoadjuvant chemotherapy and with Miller and Payne G4 or G5 and LN- status were assigned to the observation group. Distant disease-free survival was the primary endpoint. The final intention-to-treat analysis comprised 379 patients. After a median follow-up period of 72.4 months, the 5-year distant disease-free survival was 92% and 90% in the chemotherapy plus ET and ET-alone groups, respectively. Comparatively, the observation group showed a trend towards better distant disease-free survival. For patients non-responsive to neoadjuvant chemotherapy, adjuvant non-cross-resistant chemotherapy did not significantly improve distant disease-free survival compared to ET alone.

## Introduction

Previous randomized controlled trials (RCTs) have failed to illustrate a significant difference in survival outcomes between using the same chemotherapy regimen in neoadjuvant and adjuvant settings^1,2^_._ Although no survival benefit has been found with neoadjuvant chemotherapy (NCT), these trials have demonstrated that the pathological complete response (pCR) was associated with better survival. Consequently, two strategies were established to overcome potential drug resistance; the first was adding a new drug in the NCT setting to improve the pCR rate, and the second was adding a non-cross-resistant regimen in a selected population with a high recurrence risk (i.e., non-pCR patients).

Two major RCTs have explored the advantages of altering ongoing NCT after an early assessment of the initial clinical response. The GeparTrio trial adjusted the NCT regimen based on ultrasound assessment after two cycles of a combination of docetaxel (Taxotere), doxorubicin hydrochloride (Adriamycin), and cyclophosphamide (TAC therapy). All non-responsive patients were randomized to receive either four cycles of TAC or four cycles of vinorelbine plus capecitabine (NX)^3^. Patients in the study by Smith et al. ^4^ were administered NCT with four cycles of a combination of cyclophosphamide, doxorubicin, vincristine, and prednisone (CAVP), and clinical response was evaluated by physical examination after four cycles of NCT. All responsive patients were randomized to receive four additional cycles of CAVP or docetaxel, whereas non-responsive patients received four cycles of docetaxel. Both trials failed to demonstrate a significant increase in the pCR rate after modification of treatment based on clinical response in patients who were non-responsive to NCT.

Compared to clinical response, administering additional treatments with different mechanisms based on pathological response may be a reasonable therapeutic approach. Thomas et al. adapted this postoperative treatment strategy^5^. In their trial, all patients received three cycles of neoadjuvant CAVP. If the pathological response was poor, the patients were randomized to receive five cycles of CAVP or a combination of vincristine, bleomycin, methotrexate, and fluorouracil (VbMF). The patients in the VbMF group had better survival outcomes. Hormone receptor-positive (HR + )/human epidermal growth factor receptor 2-negative (HER2-) breast cancer is known to be non-responsive to chemotherapy; however, lymph node positivity (LN+ ) is an indicator for chemotherapy. Therefore, it remains controversial which chemotherapy regimen is superior and how many cycles of that regimen are appropriate for HR + /HER2-/LN+ patients. Non-cross-resistance regimens are commonly used in the treatment of advanced breast cancer^6^. However, no previous alternative non-cross-resistant adjuvant chemotherapy was established for HR+ /HER2-/LN+ patients who were non-responsive to primary chemotherapy.

A retrospective analysis conducted at our center show that for patients with HR + /HER2- breast cancer, switching to a non-cross-resistant regimen after NCT (containing anthracycline or taxane) could have better distant disease-free survival (DDFS) (unpublished data). Based on this result, we evaluate the strategy of using pathological response-guided non-cross-resistant adjuvant chemotherapy for patients with HR + /HER2-/LN+ breast cancer who are non-responsive to primary chemotherapy.

## Methods

This multi-center, randomized, controlled, phase III trial was conducted across eight hospitals located in Beijing, China. The trial was designed by the Peking University Cancer Hospital and supervised by the institutional review board at Peking University Cancer Hospital, Beijing Chao Yang Hospital, Chinese Academy of Medical Sciences, Peking University People’s Hospital, Xuan Wu Hospital Capital Medical University, 307 Hospital of PLA, and Peking University First Hospital, and was registered on 25 November 2009, at ClinicalTrials.gov, and the registration number is NCT01019616. The study was done in accordance with the principles of the Declaration of Helsinki. All written informed consent were obtained.

Eligible patients were women aged <65 years with invasive breast cancer and positive axillary lymph nodes (diagnosed by fine needle aspiration, core needle biopsy, or sentinel lymph node biopsy). Patients with estrogen receptor-positive or progesterone receptor-positive (≥10% by immunohistochemistry) and HER2- (0 or 1+ by immunohistochemistry or HER2/chromosome enumeration probe ratio <1.8 by fluorescent in situ hybridization) breast cancer were required. Patients had to complete four cycles of NCT (containing anthracycline), undergo radical surgery, and be non-responsive to NCT according to pathological assessment. An experienced pathologist employed at the Peking University Cancer Hospital who was blinded to the groupings used the Miller and Payne (M&P) grading system to assess the pathological response of the primary tumor as follows: grade 1: there was no change in individual tumor cells and no decrease in overall cellularity; grade 2: <30% necrosis of tumor cells; grade 3: 30–90% necrosis of tumor cells; grade 4: >90% necrosis of tumor cells; grade 5: no tumor cells were identified in sections obtained from the tumor site; however, ductal carcinoma in situ residue alone might be present. Non-responsiveness to treatment was defined as M&P grade 1–3 disease, or any stage with residual positive lymph nodes in the surgical specimen. The other seven hospitals each had their own pathologists perform pathological grading of the specimen.

The main exclusion criteria were a history of other malignant tumors, metastatic breast cancer, and any clinically serious medical conditions.

### Randomization and treatment

All intention-to-treat (ITT) patients were enrolled preoperatively, and the eligible patients were randomly assigned at a 1:1 ratio within 4 weeks postoperatively. The other ITT patients who did not meet the pathological non-responsiveness criteria were assigned to the observation group. The patients in arm A received four cycles of a non-cross-resistant regimen plus endocrine therapy (ET), while the patients in arm B patients received ET alone. The randomization sequence was created using SPSS software (version 11.0; IBM Corp., Armonk, NY, USA) and was stratified based on NCT regimens (anthracycline- or taxane-based therapy vs. concurrent anthracycline and taxane usage) and pathological response (M&P staging G1 and G2 vs. M&P staging G3) using random block sizes of four. Patients with M&P G4–5 disease and residual positive lymph nodes were assigned to the M&P G3 subgroup. For patients with limited lymph node metastasis (one or two diagnosed by sentinel lymph node biopsy) after undergoing breast-conserving surgery, we omitted axillary lymph node dissection.

Patients enrolled from Beijing Cancer Hospital started with four cycles of CEF3w (cyclophosphamide 600 mg/m^2^ on day 1; epirubicin 90–100 mg/m^2^ on day 1; 5-fluorouracil 600 mg/m^2^ on day 1, every 3 weeks), and after being assigned to the chemotherapy group, they received four cycles of Tq1w (paclitaxel 80 mg/m^2^ on days 1, 8, and 15, every 3 weeks) or TPq1w (paclitaxel 80 mg/m^2^ on days 1, 8, and 15 and carboplatin AUC 6 on day 1, every 3 weeks). Patients from the other hospitals started with four cycles of TAC (docetaxel 75 mg/m^2^, doxorubicin 50 mg/m^2^, and cyclophosphamide 500 mg/m^2^, every 3 weeks) or TE (docetaxel 75 mg/m^2^ and epirubicin 75 mg/m^2^, every 3 weeks) and received four cycles of adjuvant chemotherapy with NX (vinorelbine 25 mg/m^2^ on days 1 and 8 plus capecitabine 1000 mg/m^2^ orally twice/day on days 1–14, every 3 weeks) or NP (vinorelbine 25 mg/m^2^ on days 1 and 8 and carboplatin AUC 6 on day 1, every 3 weeks) when assigned to the chemotherapy group. Patients in both arms received tamoxifen or aromatase inhibitors for 5 years and underwent whole breast irradiation after breast-conserving surgery, or chest wall and supraclavicular region irradiation after mastectomy, before 2014. After the results of the SOFT and TEXT trials were published (2014), the doctors decided on the use of gonadotropin-releasing hormone agonists for premenopausal patients with breast cancer. The adjuvant treatment regimen for patients in the observation group was also determined by doctors, with four cycles of non-cross-resistant chemotherapy plus ET or ET alone being prescribed. All enrolled patients were followed up postoperatively every 3 months over the first 2 years, every 6 months over the next 3–5 years, and once a year for 5 years after that.

### Endpoint assessment

The primary endpoint was DDFS, defined as the interval between the date of surgery and the occurrence of the first distant event or breast cancer-specific death, whichever occurred first. The secondary endpoints were invasive disease-free survival (iDFS) and overall survival (OS). Liver metastases, loco-regional metastases, contra-lateral breast cancer, and second primary cancer were diagnosed based on pathology, while brain, bone, and lung metastases were diagnosed based on radiology.

### Statistical analysis

A sample of 350 patients (ET-alone group: *n* = 175; non-cross-resistant chemotherapy plus ET group: *n* = 175) was planned due to a requirement of 74 distant disease events to provide 80% power to detect a hazard ratio (HR) of 0.75 with a two-sided significance level of 5% in the primary analysis. According to the retrospective data available at our center, we assumed that the DDFS at 5 years was 88% in the non-cross-resistant adjuvant chemotherapy group and 73% in the ET-alone group. Because the median follow-up time at the time of writing was 72.4 months, the DDFS event rates were substantially lower than originally expected, and since an additional delay of 5–8 years was considered to be unacceptably long, we deduced that “time-driven” rather than “event-driven” analyses would be more appropriate.

**Fig. 1:**
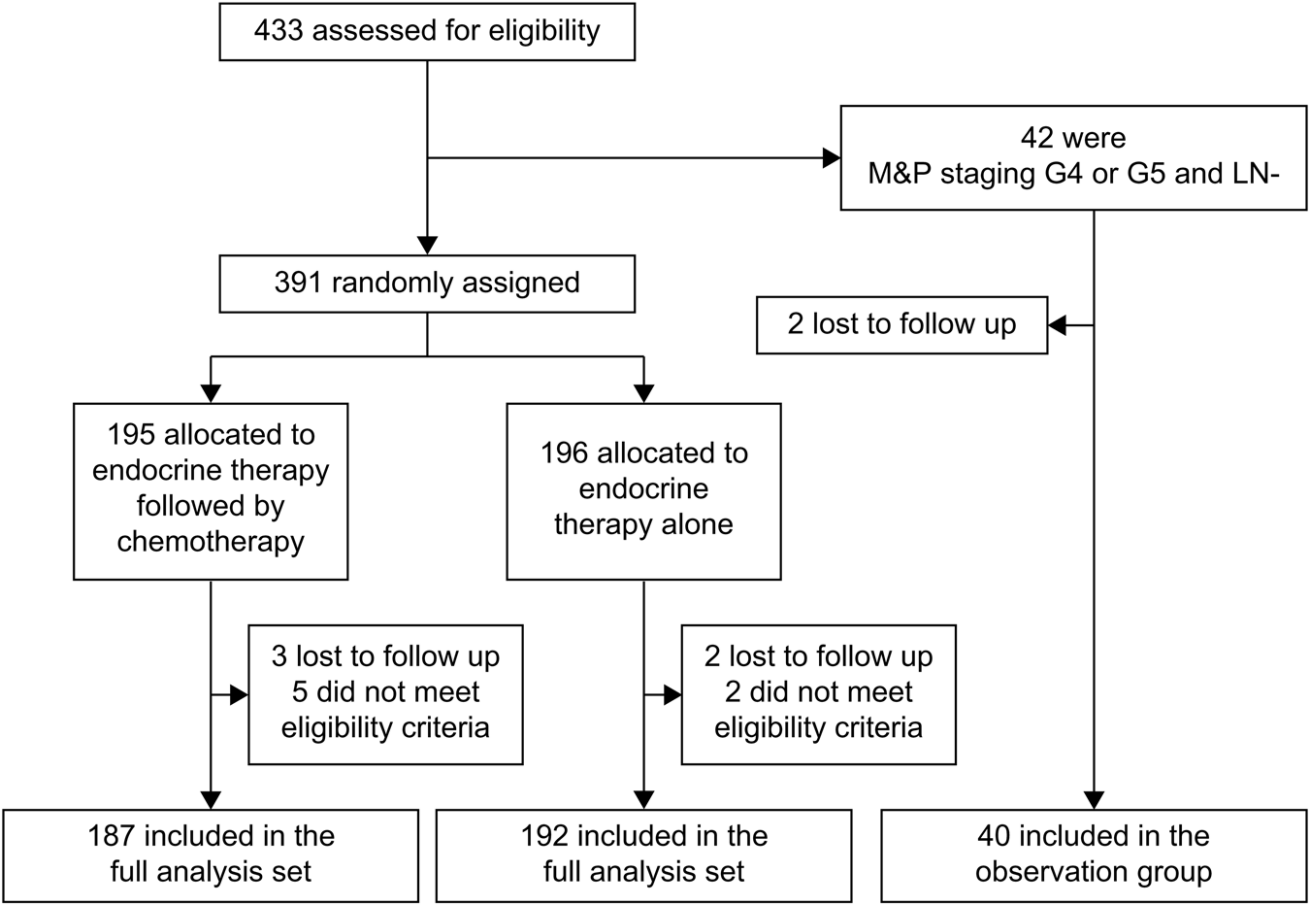
CONSORT diagram. M&P Miller and Payne, LN lymph node-negative.

All cases were analyzed on an ITT basis, including patients in the observation group (Fig. 1). Time-to-event endpoints were calculated using the Kaplan‒Meier method and compared between patient groups using the log-rank test. HRs and 95% confidence intervals (95% CIs) were estimated using a Cox proportional hazards regression model. A two-sided *P*-value < 0.05 was considered statistically significant.

### Reporting summary

Further information on research design is available in the Nature Portfolio Reporting Summary linked to this article.

## Results

From October 2010 to September 2018, 433 patients were enrolled in the present trial. From this group, 195 patients were randomly assigned to receive four cycles of non-cross-resistant chemotherapy plus ET, while 196 patients were assigned to receive ET alone across the eight participating hospitals located in Beijing. Forty-two of these patients were diagnosed with M&P G4–5 disease but no pathological lymph nodes. Fourteen patients were excluded from the final analysis, seven patients were lost to follow-up, and the others did not meet the eligibility criteria (i.e., four patients had triple-negative breast cancer, and three had HER2+ breast cancer). Therefore, the final ITT analysis comprised 379 patients (Chemotherapy plus ET group: *n* = 187; ET-alone group: *n* = 192) in the two randomized groups and 40 patients in the observation group.

### Baseline characteristics

Table 1 presents the baseline characteristics of the patient sample. All patients had stage IIB, IIIA, or IIIB breast cancer. In the two randomized groups, 89.1% of patients received four cycles of the CEF regimen as NCT, while the others received four cycles of concurrent regimens (TAC or TE) as NCT. The pathological evaluation in 40.9% of patients was M&P G1 and G2, 53% were M&P G3, and 6.1% were M&P G4 or 5. Moreover, positive lymph nodes were found in 67.7% of patients after NCT. The baseline characteristics of the two groups were balanced. The observation group included 9.5% of cases; patients in this group had a higher Ki67 rate and were more responsive to NCT according to clinical response; all patients were M&P G4 or G5 according to pathological evaluation, and only one patient had a positive lymph node after NCT (Table 1).

**Table 1:** Basic characteristics.

	ET followed by CT	ET alone	Observation group
	*N* = 187	*N* = 192	*N* = 40
Age, years			
Median (range)	48 (27‒72)	47 (24‒73)	47.8 (33‒71)
Menopausal status			
Yes	49	52	9
No	138	140	31
Tumor size			
1	38	36	9
2	135	139	30
3	14	17	1
Histological type			
IDC	177	183	36
ILC	8	8	4
Mucinous tumor	2	1	0
Ki67			
≤14%	50	59	3
≥15%	137	133	37
Lymph node diagnostic method			
CNB or FNA	122	125	24
SLNB	65	67	16
NCT regimen			
CEF	169	175	36
TAC or TE	18	17	4
Clinical response			
uCR	0	0	5
uPR	87	90	27
uSD	100	102	8
Surgery			
Mastectomy	136	138	24
Conserving surgery	51	54	16
M&P staging			
1 or 2	80	77	0
3	96	105	0
4 or 5	11	10	40
No. of lymph nodes involved after NCT			
ypN0	51	49	39
ypN1	81	79	1
ypN2	49	54	0
Unknown	6	10	0
CPS + EG score			
1 or 2 with ypN0	54	53	39
3 or 2 with ypN+	149	157	1
Unknown	6	10	0
ET regimen			
TAM	36	34	8
AI	138	140	31
AI + OFS	13	18	1

*ET* endocrine therapy, *CT* chemotherapy, *IDC* invasive ductal cancer, *ILC* invasive lobular cancer, *CNB* core needle biopsy, *FNA* fine needle aspiration, *SLNB* sentinel lymph node biopsy, *NCT* neoadjuvant chemotherapy, *CEF* cyclophosphamide, epirubicin, 5-fluorouracil, *TAC* docetaxel, doxorubicin, cyclophosphamide, *TE* docetaxel, epirubicin, *uCR* ultrasound complete response, *uPR* ultrasound partial response, *uSD* ultrasound stable disease, *M&P staging* Miller and Payne staging, *CPS* *+* *EG* clinical, pathological stage, estrogen receptor, grading, *TAM* tamoxifen, *AI* aromatase inhibitor, *OFS* ovarian function suppression.

### First endpoint

**Fig. 2:**
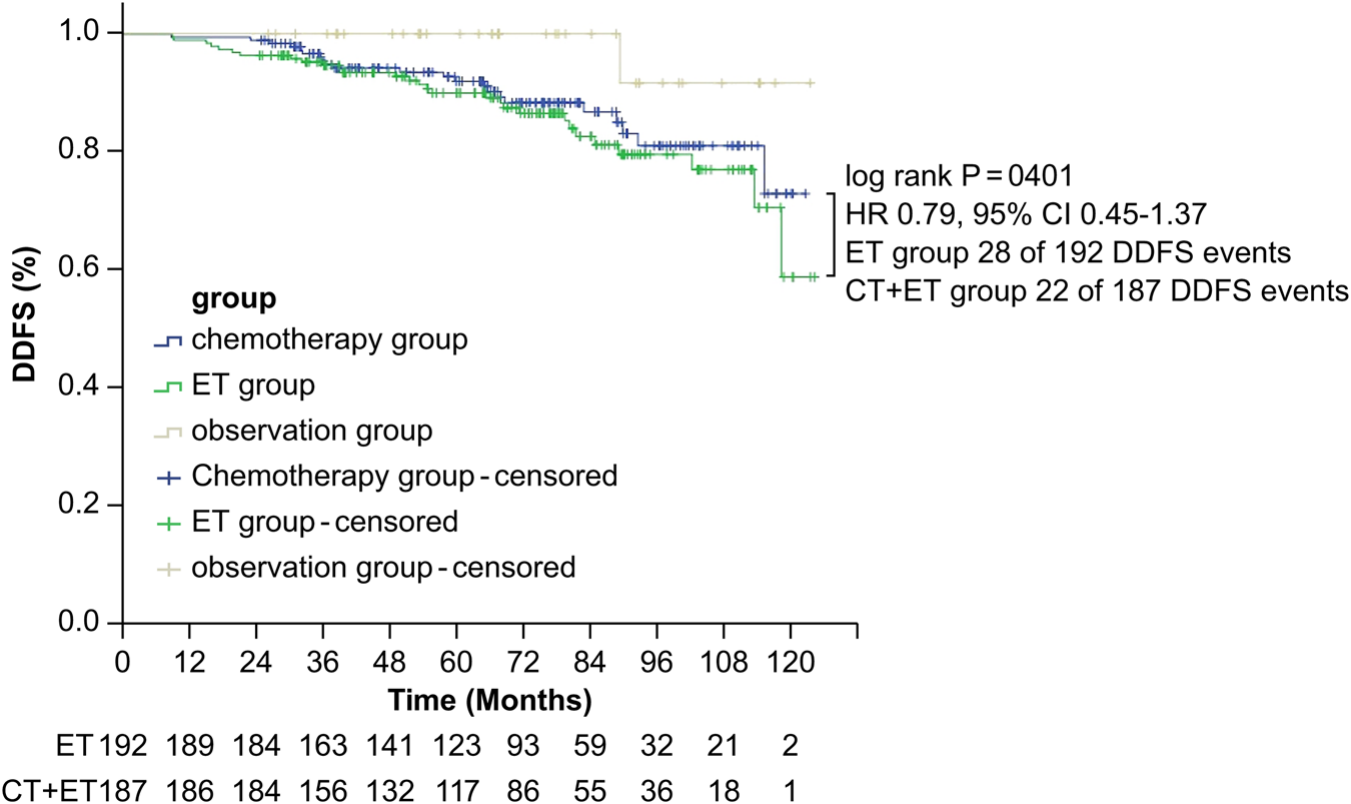
Kaplan‒Meier estimates for DDFS. DDFS distant disease-free survival, CT chemotherapy, ET endocrine therapy, HR hazard ratio.

**Fig. 3:**
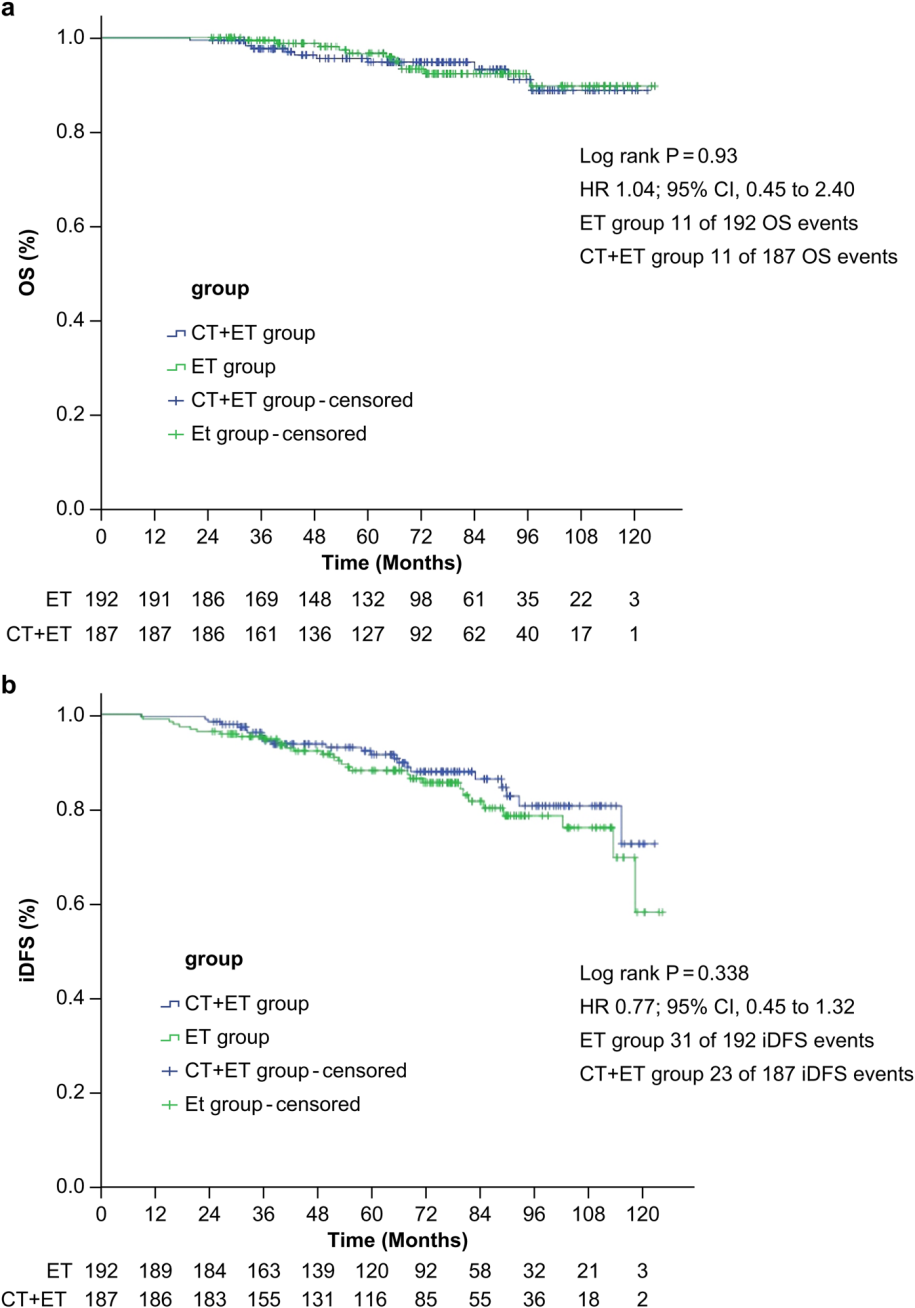
Kaplan‒Meier estimates for OS. (**a**) and Kaplan‒Meier estimates for iDFS (**b**). OS overall survival, iDFS invasive disease-free survival, CT chemotherapy, ET endocrine therapy, HR hazard ratio.

Only 50 patients developed distant disease after a median follow-up of 72.4 months. DDFS events occurred in 22 patients in the chemotherapy plus ET group (11.7%) and in 28 patients in the ET-alone group (14.6%). These events are summarized in Table 2. The 5-year DDFS was 92% (95% CI, 88%–96%) in the chemotherapy plus ET group and 90% (95% CI, 86%–94%) in the ET-alone group (HR 0.79, 95% CI 0.45‒1.37) (*P* = 0.401; Fig. 2). No significant improvement in DDFS was found in the chemotherapy plus ET groups in the prespecified NCT regimen and M&P staging subgroups (Fig. 3).

**Table 2:** Site of first distance disease-free survival events.

	ET followed by CT	ET alone	Observation	Overall
Site of first DDFS event	*N* = 22	*N* = 28	*N* = 1	*N* = 51
Bone	4	12	0	16
Lung	2	3	0	5
Liver	0	4	0	4
Pleura	3	1	0	4
Brain	1	0	0	1
Multiple distant metastases	11	5	1	17
Death without event	1	3	0	4

*DDFS* distant disease-free survival, *ET* endocrine therapy, *CT* chemotherapy.

### Exploratory analysis

**Fig. 4:**
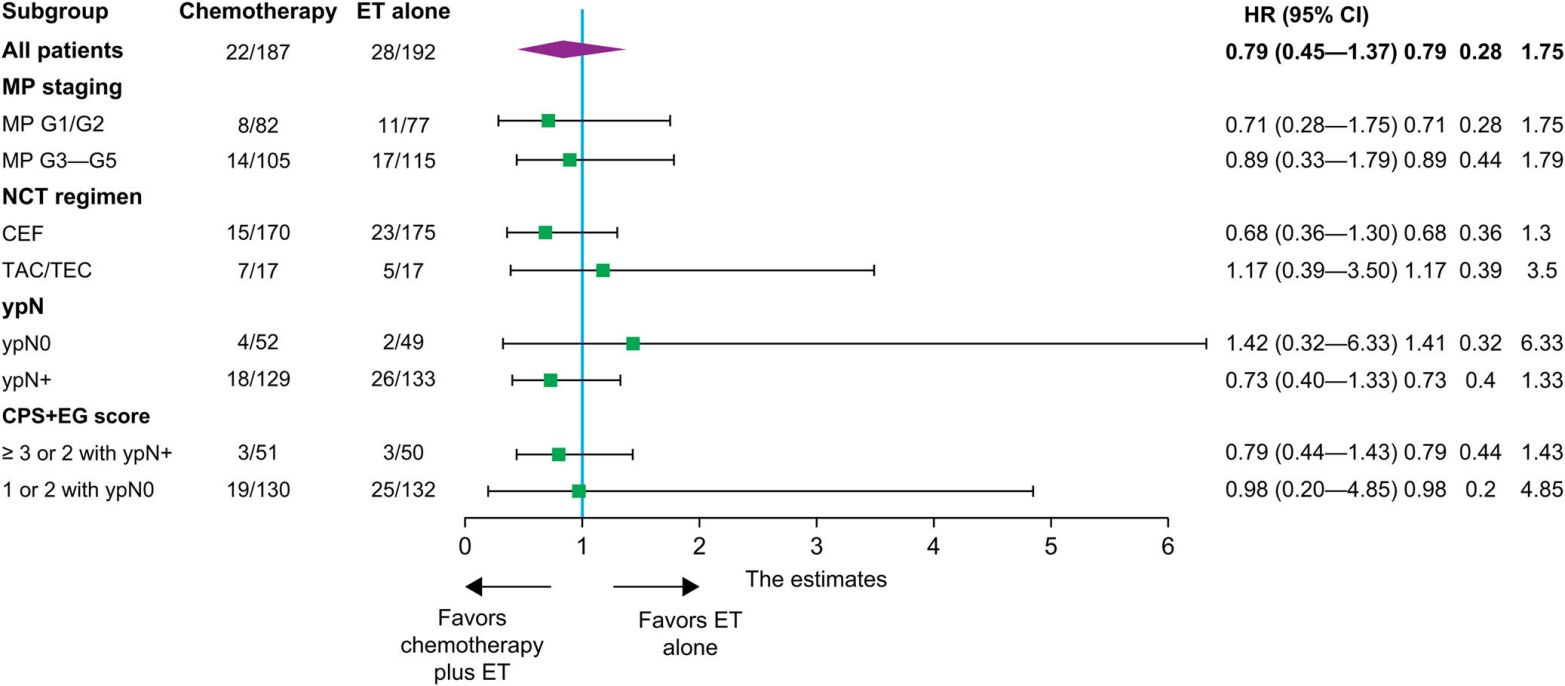
Forest plot of univariable Cox regression for DDFS in subgroups. DDFS distant disease-free survival, CT chemotherapy, ET endocrine therapy, HR hazard ratio, M&P Miller and Payne, CPS-EG clinical, pathological stage, estrogen receptor, grading, NCT neoadjuvant chemotherapy, CEF cyclophosphamide, epirubicin, 5-fluorouracil, TAC docetaxel, doxorubicin, cyclophosphamide, TE docetaxel, epirubicin, ypN0 yield pathological lymph node-negative, ypN+ yield pathological lymph node-positive.

In a further exploratory analysis, we stratified the patients into ypN0 or ypN+ subgroups. These subgroups were further divided into patients with a clinical pathological stage plus estrogen receptor (ER) status and nuclear grading (CPS + EG) score of 1 or 2 for the ypN0 group and a CPS + EG score of 3 or 2 for the ypN+ group. Non-cross-resistant chemotherapy did not improve survival in these subgroups. In the ypN+ subgroup, compared to the ET-alone group, we found a 7% absolute benefit for 5-year DDFS in the non-cross-resistant chemotherapy plus ET group (90.2% vs. 83.2%, HR 0.73, 95% CI: 0.4‒1.33; *P* = 0.196) (Fig. 4). In the observation group, only one DDFS event was observed. Patients in the observation group had a trend towards better DDFS compared to the two randomized groups (*P* = 0.107) (Fig. 2). However, this trend was not observed for OS.

### Secondary endpoints

The secondary endpoints were OS and iDFS. Overall, 22 deaths were reported (11 in the non-cross-resistant chemotherapy plus ET group and 11 in the ET-alone group). Both OS (HR 1.04; 95% CI, 0.45‒2.40) and iDFS (HR 0.77; 95% CI, 0.45‒1.32) differed significantly between the two groups (Fig. 3).

## Discussion

There are few studies focus on post-neoadjuvant treatment in HR + /HER2-/LN+ breast cancer. We previously retrospectively analyzed patients with HR + /HER2- breast cancer who were diagnosed before 2007 and had received two to four cycles of NCT (containing anthracycline or taxane). After a median follow-up of 44 months, we observed significantly better DDFS in patients who received non-cross-resistant chemotherapy plus ET after surgery compared to those who received ET alone (124 vs. 75 months, *P* = 0.049) (unpublished data). To evaluate the strategy of using pathological response-guided non-cross-resistant adjuvant chemotherapy for HR + /HER2-/LN+ breast cancer patients who were non-responsive to NCT, we started this trial in 2010. There had been no previous phase III RCT that attempted to demonstrate a post-neoadjuvant treatment strategy in patients with HR+ /HER2-/LN+ status. After a median follow-up of 72.4 months, the 5-year DDFS rate in our trial was 92% (95% CI, 88–96) in the non-cross-resistant adjuvant chemotherapy plus ET group and 90% (95% CI, 86–94) in the ET-alone group (HR 0.79, 95% CI 0.45‒1.37; *P* = 0.401).

Similar to previous studies, our trial failed to demonstrate a survival benefit for additional non-cross-resistant adjuvant chemotherapy in non-responsive patients with HR + /HER2-/LN+ breast cancer. The absolute benefit for the 5-year DDFS was 2% in our trial in the additional chemotherapy group, compared to 3% in the HR+ subgroup in the CREATE-X trial. There are several explanations for our findings. First, HR + /HER2-/LN+ patients might also be non-responsive to additional non-cross-resistant chemotherapy; therefore, enhanced chemotherapy did not improve survival. Second, 73% of all patients were post-menopausal, and 31% of premenopausal patients had used drugs that suppressed ovarian function; these features might affect the effectiveness of chemotherapy. Third, the chemotherapy regimens in the sub-sites were different from that in our center; each sub-site used its own pathologist to evaluate the pathological response. These confounding factors might have affected the results of our study. In the survival analysis of the prespecified subsite group (TAC/TE group), there was an intersection of the survival curves between the non-cross-resistant chemotherapy group and the ET-alone group. Only one multi-center, open-label, randomized, controlled, phase III trial (POTENT) had shown survival improvement when using additional chemotherapy in patients with HR+/HER2-/LN+ breast cancer. This trial included 392 (20%) patients who received NCT, and adjuvant S1 significantly improved the 5-year iDFS (HR 0.66) in this subgroup^6^. Therefore, the role of postoperative chemotherapy in HR+/HER2-/LN+ patients remains controversial.

An association between pCR, recurrence-free survival, and OS in patients with breast cancer was found in a previous pooled analysis^7^. Partial drug resistance in tumors could be indicated by the presence of residual disease (non-pCR) after NCT. The administration of non-cross-resistant adjuvant treatment based on pathological response could potentially overcome drug resistance. The CREATE-X trial administered non-cross-resistant adjuvant treatment to non-pCR patients with HER2- breast cancer, while the KATHERINE trial used this treatment in non-pCR patients with HER2+ breast cancer. The role of post-neoadjuvant treatment was highlighted in triple-negative and HER2+ breast cancer^8,9^. However, this strategy of using non-cross-resistant chemotherapy in non-pCR patients was not validated in the HR+ subgroup in the CREATE-X trial, and the absolute benefit in terms of DFS and OS was only 3% and 3.4%, respectively, in the capecitabine group^8^. It is possible that this is due to the significant tumor heterogeneity between triple-negative, HER2+ breast cancer and HR+/HER2- breast cancer, which is non-responsive to chemotherapy. The pCR rate is significantly lower after NCT for HR+/HER2- breast cancer. The CTNeoBC pooled analysis showed that the pCR rate was only 9.6% in HR+/HER2- patients, which was much lower than that in patients with HER2+ and triple-negative breast cancer^7^. The pCR rate of patients in this study was even lower at 4.7%, suggesting that the patients in this study may be insensitive to both NCT and non-cross-resistant adjuvant chemotherapy. Moreover, whether the benefit of chemotherapy in patients with HR+/HER2− breast cancer stems from the cytotoxicity of chemotherapy or chemotherapy-induced amenorrhea is inconclusive; only 26.6% of patients in this study were premenopausal, 30.6% of whom also underwent ovarian function suppression therapy, which may have further diluted the benefit generated by non-cross-resistant chemotherapy. Therefore, we did not use non-pCR as an indicator to administer additional post-neoadjuvant treatment in this study. We defined M&P G1 to G3, or any grade of M&P staging with pathological lymph nodes, in surgical specimens as cancer non-responsive to NCT. Besides our non-responsive criterion for patients with HR+/HER2− breast cancer, Mittendorf et al. developed the CPS + EG staging system in 2008 and validated this staging system in two independent cohorts in 2011, 1 year after the initiation of our trial^10,11^. Patients with a CPS + EG score ≥3 or 2 and pathological lymph nodes had worse DFS. In our study, 81% of patients were CPS + EG score ≥3 or 2 and had pathological lymph nodes. Therefore, we believe that using different non-responsive criteria would not affect our results.

In the exploratory analysis of our trial, we found a 7% absolute DDFS benefit in patients with pathological lymph nodes (69.3%) in the chemotherapy plus ET group (*P* = 0.196). Further studies are needed to demonstrate this benefit in patients with pathological lymph nodes.

In the present study, 9.2% of patients were responsive to NCT (observation group). When comparing the two randomized groups and the observation group, patients in the observation group showed a trend towards better DDFS (*P* = 0.107; Fig. 2). After a median follow-up of 72.4 months, only one of the 40 patients in the observation group had a DDFS event. Thus, four cycles of NCT plus ET might be considered sufficient for these patients.

Another study with an enrolled population of HR+/HER2-/LN+ breast cancer patients is RxPONDER which failed to demonstrate an iDFS benefit with the application of chemotherapy in patients with a 70-gene test score of 0–25. However, patients in the prespecified premenopausal subgroup had a longer iDFS in the chemotherapy group than those in the ET alone group. The population enrolled in this study had a more advanced clinical stage, with 27.2% of patients having ypN2, and all enrolled patients were not responsive to NCT. From the results of this study, the survival benefit of non-cross-resistant adjuvant chemotherapy is limited; therefore, adjuvant ET might remain the mainstay in patients with this type of breast cancer.

Besides chemotherapy, cyclin-dependent kinase (CDK) inhibitors have been administered to patients with high-risk HR + /HER2- early breast cancer in some trials. The Monarch E and PALLAS trials showed different results when administering additional adjuvant CDK4/6 inhibitors for 2 years in the afore mentioned patient groups^12,13^. Abemaciclib combined with ET achieved better iDFS compared to ET alone in the Monarch E trial, while palbociclib plus ET did not achieve better iDFS compared to ET alone in the PALLAS trial. PENELOPE-B was the first trial to administer palbociclib for 1 year after NCT and surgery, and patients with CPS + EG scores of ≥ 2 along with pathological lymph nodes in that trial were randomized to the ET or ET plus palbociclib groups. After a median follow-up of 43 months, 1-year additional palbociclib administration did not achieve better iDFS in the PENELOPE-B trial. Other post-neoadjuvant treatment strategies include poly (adenosine diphosphate–ribose) polymerase inhibitors. In OlympiA trial, there were 168 HR + HER2- patients in the olaparib group, of whom 104 received NCT, and the absolute benefit of iDFS compared to the control group was 6.3% and 19%, respectively, neither of which reached statistical difference^14^.

The main limitation of the trial is the unexpectedly low rate of distant recurrences. According to the data from our center before 2007, we assumed that the 5-year DDFS rate would be 88% in the non-cross-resistant adjuvant chemotherapy plus ET group and 73% in the ET-alone group. However, in this trial, the 5-year DDFS rate is 92% in the chemotherapy group and 90% in the ET group. We only found trends towards better DDFS in patients with pathological lymph nodes. We need a larger sample size to make exactly this difference. Further studies should be performed on patients with pathological node-positive breast cancer. The second limitation is that HR + HER2- breast cancer may not be a good candidate for enhanced chemotherapy treatment. Other post-neoadjuvant treatment regimens including abemaciclib and olaparib have yielded better results, and these drugs may be the direction for enhanced treatment of HR+HER2-LN+ breast cancer patients.

In conclusion, this is the first prospective phase III randomized trial to validate the benefit of switching to a non-cross-resistant regimen after NCT for non-responsive patients with HR + /HER2-/LN+ breast cancer. However, additional non-cross-resistant adjuvant chemotherapy did not improve survival in non-responsive HR+/HER2-/LN+ patients. A trend towards better DDFS was observed in patients who were responsive to NCT; thus, four cycles of NCT (containing anthracycline) might be considered sufficient for these patients.

## Supplementary information

Reporting summary

## Data Availability

The datasets generated during and/or analyzed during the current study are available from the corresponding author on reasonable request.

## Code availability

The data that support the findings of this study are available on request from the corresponding author. The data publicly available due to privacy or ethical restrictions.
