# Author Correction: Phase III study of HR-positive/HER2-negative/lymph node-positive breast cancer non-responsive to primary chemotherapy: a randomized trial

**DOI:** 10.1038/s41523-023-00567-6

**Published:** 2023-07-24

**Authors:** Yang Yang, Yingjian He, Zhaoqing Fan, Xue Chen, Yiqiang Liu, Chao Zhang, Hongchuan Jiang, Xin Wang, Xiang Wang, Fei Xie, Shu Wang, Bin Luo, Hua Kang, Tao Wang, Zefei Jiang, Peng Yuan, Binhe Xu, Ling Xu, Yinhua Liu, Jinfeng Li, Yuntao Xie, Tianfeng Wang, Tao Ouyang

**Affiliations:** 1 Key Laboratory of Carcinogenesis and Translational Research (Ministry of Education), Breast Center, Peking University Cancer Hospital & Institute, Beijing, China; 2 Beijing Chao Yang Hospital, Breast Disease Department, Beijing, China; 3 Cancer Hospital, Chinese Academy of Medical Sciences, Breast Cancer Department, Beijing, China; 4 Peking University People’s Hospital, Breast Cancer Center, Beijing, China; 5 Beijing Tsinghua Changgeng Hospital, Breast Disease Department, Beijing, China; 6 Xuan Wu Hospital Capital Medical University, Breast Disease Department, Beijing, China; 7 307 Hospital of PLA, Breast Cancer Department, Beijing, China; 8 Peking University First Hospital, Breast Cancer Center, Beijing, China

**Keywords:** Randomized controlled trials, Cancer therapy

Correction to: *npj Breast Cancer* 10.1038/s41523-023-00553-y, published online 21 June 2023

“In this article the affiliation details for Author’s Jinfeng Li, Yuntao Xie, and Tianfeng Wang were incorrectly given as ‘Cancer Hospital, Chinese Academy of Medical Sciences, Breast Cancer Department, Beijing, China’ but should have been ‘Key Laboratory of Carcinogenesis and Translational Research (Ministry of Education), Breast Center, Peking University Cancer Hospital & Institute, Beijing, China’. The original article has been corrected.“

